# ChatGPT Performance in Diagnostic Clinical Microbiology Laboratory-Oriented Case Scenarios

**DOI:** 10.7759/cureus.50629

**Published:** 2023-12-16

**Authors:** Malik Sallam, Khaled Al-Salahat, Eyad Al-Ajlouni

**Affiliations:** 1 Department of Pathology, Microbiology and Forensic Medicine, The University of Jordan, School of Medicine, Amman, JOR; 2 Department of Clinical Laboratories and Forensic Medicine, Jordan University Hospital, Amman, JOR

**Keywords:** healthcare practice, ai chatbot gpt-4, applications of ai, medical and diagnostic microbiology, clinical laboratory

## Abstract

Background: Artificial intelligence (AI)-based tools can reshape healthcare practice. This includes ChatGPT which is considered among the most popular AI-based conversational models. Nevertheless, the performance of different versions of ChatGPT needs further evaluation in different settings to assess its reliability and credibility in various healthcare-related tasks. Therefore, the current study aimed to assess the performance of the freely available ChatGPT-3.5 and the paid version ChatGPT-4 in 10 different diagnostic clinical microbiology case scenarios.

Methods: The current study followed the METRICS (Model, Evaluation, Timing/Transparency, Range/Randomization, Individual factors, Count, Specificity of the prompts/language) checklist for standardization of the design and reporting of AI-based studies in healthcare. The models tested on December 3, 2023 included ChatGPT-3.5 and ChatGPT-4 and the evaluation of the ChatGPT-generated content was based on the CLEAR tool (Completeness, Lack of false information, Evidence support, Appropriateness, and Relevance) assessed on a 5-point Likert scale with a range of the CLEAR scores of 1-5. ChatGPT output was evaluated by two raters independently and the inter-rater agreement was based on the Cohen’s κ statistic. Ten diagnostic clinical microbiology laboratory case scenarios were created in the English language by three microbiologists at diverse levels of expertise following an internal discussion of common cases observed in Jordan. The range of topics included bacteriology, mycology, parasitology, and virology cases. Specific prompts were tailored based on the CLEAR tool and a new session was selected following prompting each case scenario.

Results: The Cohen’s κ values for the five CLEAR items were 0.351-0.737 for ChatGPT-3.5 and 0.294-0.701 for ChatGPT-4 indicating fair to good agreement and suitability for analysis. Based on the average CLEAR scores, ChatGPT-4 outperformed ChatGPT-3.5 (mean: 2.64±1.06 vs. 3.21±1.05, *P*=.012, t-test). The performance of each model varied based on the CLEAR items, with the lowest performance for the “Relevance” item (2.15±0.71 for ChatGPT-3.5 and 2.65±1.16 for ChatGPT-4). A statistically significant difference upon assessing the performance per each CLEAR item was only seen in ChatGPT-4 with the best performance in “Completeness”, “Lack of false information”, and “Evidence support” (*P*=0.043). The lowest level of performance for both models was observed with antimicrobial susceptibility testing (AST) queries while the highest level of performance was seen in bacterial and mycologic identification.

Conclusions: Assessment of ChatGPT performance across different diagnostic clinical microbiology case scenarios showed that ChatGPT-4 outperformed ChatGPT-3.5. The performance of ChatGPT demonstrated noticeable variability depending on the specific topic evaluated. A primary shortcoming of both ChatGPT models was the tendency to generate irrelevant content lacking the needed focus. Although the overall ChatGPT performance in these diagnostic microbiology case scenarios might be described as “above average” at best, there remains a significant potential for improvement, considering the identified limitations and unsatisfactory results in a few cases.

## Introduction

The utility of artificial intelligence (AI) in healthcare marks a transformative era in diagnostics approaches in healthcare [1,2]. This involves more efficient laboratory processes with improved workflow, patient care, and satisfaction [2,3]. Among the AI-based conversational models that could create such a transformative change is the Chat Generative Pre-trained Transformer (ChatGPT; OpenAI, San Francisco, CA) launched publicly on November 30, 2022 [4,5]. However, the successful implementation of AI-based models in healthcare requires more extensive and robust studies to assess the performance of these models, their reliability, and possible shortcomings [2,6].

Despite the great potential and perspectives of AI-based models such as ChatGPT in healthcare, one of the challenges that needs to be investigated is the variability in performance across different tested subjects [7-11]. This variability in performance across various medical fields can be related to several factors. For example, the quality of training data is an important determinant of AI-based model performance with below-bar performance in rare medical conditions [12]. Another factor is the architecture of various AI-based models where image analysis and processing of text data may vary [13]. Additionally, the regulatory and standardization guidelines of various medical fields can change at a rate beyond the limit of knowledge of AI-based models [14]. Furthermore, the aforementioned standards and guidelines might not be easily accessible for training of the AI-based models with subsequent effect on the performance of these models (e.g., the Clinical and Laboratory Standards Institute (CLSI) antimicrobial susceptibility testing (AST) standards).

In medical practice, certain specialties are particularly considered in prime position to benefit from AI integration driving enthusiasm regarding the potential improvements in the workflow [2,15]. Nevertheless, concerns regarding potential job loss due to AI automation are also notable [15]. The AI-based models have the ability to efficiently process and analyze extensive datasets [2,6]. Therefore, radiology and pathology (with clinical pathology included) where precision and quick turnaround times are critical can be viewed as the leading specialties to benefit from the AI transformation [16,17]. This capability has significant implications for the practice of clinical microbiology, where AI integration could transform the approaches to diagnosis and treatment of infectious diseases, ultimately leading to improved patient care [18].

A recently conceived checklist for standardizing the design and reporting of AI-based studies in healthcare is called “METRICS” (Model, Evaluation; Timing/Transparency; Range/Randomization; Individual factors; Count; Specificity of the prompts/language) [19]. This framework considers the features of the tested model, the evaluation approach, and characteristics of datasets used to create the queries on the AI model [19]. Additionally, a newly designed tool termed “CLEAR” (Completeness; Lack of false information; Evidence support; Appropriateness; and Relevance) is specifically tailored to standardize the evaluation approach of the AI-model-generated content [20].

Based on the aforementioned points, the aim of this study was to assess ChatGPT performance in the setting of different diagnostic clinical microbiology laboratory case scenarios. The insights that could be gained from this study can contribute to the growing knowledge base on AI-based models’ role in healthcare with an in-depth understanding of the strengths and limitations of ChatGPT in the field of clinical microbiology.

## Materials and methods

Study design

The current study design followed a standardized approach for design and reporting for the assessment of ChatGPT performance in diagnostic clinical microbiology laboratory case scenarios. The full details of the study design based on the METRICS checklist are illustrated in the sub-sections below [19]. The evaluation of ChatGPT-generated content was based on the CLEAR tool [20]. Ethical permission was waived based on the absence of human or animal participants or experiments.

The framework for conducting this study was based on an attempt to maintain rigor and standardization in the evaluation of ChatGPT in healthcare. Thus, the current study utilized a recently devised checklist termed the “METRICS” (Model, Evaluation; Timing/Transparency; Range/Randomization; Individual factors; Count; Specificity of the prompts/language) checklist [19]. The METRICS checklist offers a detailed framework for standardizing the design and reporting of studies evaluating AI-based models’ performance in healthcare domains [19]. The approach involves careful consideration of the following factors in the design and reporting of AI-based content evaluation in healthcare: (1) the exact AI model used and its settings, (2) the evaluation approach; (3) the exact time of testing the AI model and transparency regarding the sources of queries; (4) the range of health topics tested and the randomization process for selecting the queries; (5) individual factors in the selection of queries and subjective evaluation of the AI-model output; (6) count of queries reflecting the sample size; and (7) the specificity of the prompts used to generate the output and the exact language used [19].

Additionally, the current study employed the CLEAR (Completeness; Lack of false information; Evidence support; Appropriateness; and Relevance) tool, which provides a solid basis for subjective evaluation of the AI-model-generated output [20]. The CLEAR tool was conceived to standardize assessment of AI-based model output by taking into consideration the following specific points: (1) Completeness of the generated content; (2) Lack of false information in the generated content; (3) Evidence supporting the generated content; (4) Appropriateness of the generated content in terms of being easy to follow, concise, unambiguous, and well-organized; and (5) Relevance of the content with regards to being focused without irrelevant content [20].

AI model used, timing of model testing, count of queries, and specificity of prompt/language

This study utilized two versions of ChatGPT; GPT-3.5 version, available publicly for free, and the advanced GPT-4 version, accessible through a paid service. Testing of the two models was done under their respective default configurations to ensure replicability of the generated content. Testing of both ChatGPT models was conducted on December 3, 2023, within a concise window from 10:00 to 10:30 AM, Amman, Jordan local time.

The study involved the execution of 10 distinct queries on each ChatGPT model. This number was chosen a priori to allow a practical yet thorough analysis of each query. This approach was selected to allow a more focused approach in both the generation and evaluation of the AI-generated responses, without the need to extend the time for conducting the queries based on the rate limits of ChatGPT.

For each query, ChatGPT was prompted with a consistent and specific opening statement: “Act as an experienced microbiologist and provide a complete, accurate, evidence-based, appropriate, and relevant answer to the following query”. This standardized approach was maintained throughout all queries on both models. To prevent any potential learning or feedback loop affecting the models’ algorithms, a “New Chat” was selected before each new query, and the option “regenerate response” was not used. The prompting approach was based on the tutorial by Meskó [21]. All queries were conducted in English and the exact phrasing for each query is outlined in a public data repository “ChatGPT Performance in Clinical Microbiology Laboratory-Oriented Scenarios” (https://doi.org/10.17605/OSF.IO/92UVZ).

Individual involvement in query generation and evaluation of the ChatGPT output

The queries were derived from case scenarios created by a collaborative effort of the three authors: a consultant, a specialist, and a senior resident, all in clinical pathology/microbiology and immunology. These case scenarios were created by the three authors from the beginning without copyright issues. These cases were based on typical encounters in clinical microbiology laboratories in Jordan and involved a range of sub-specialties including bacteriology, parasitology, mycology, and virology. The content was classified loosely into three main topics: first, microbial identification techniques (biochemical, molecular, microscopic); second, AST, with a particular focus on the issues of intrinsic bacterial resistance to antimicrobials and the gold standard methods for AST; and third, the diagnostic approaches in clinical microbiology with a special focus on the need to pay attention to the quality control (QC) issues, critical result reporting, and laboratory safety protocols. The finalized form of the queries involved a subjective element since these queries were finalized based on internal discussions among the three authors-a consultant, a specialist, and a senior resident-each with varying levels of expertise in microbiology and immunology. Assessment of the generated ChatGPT content was conducted by the first and second authors independently (rater 1 and rater 2, respectively). Both raters are certified in clinical pathology/microbiology and immunology by the Jordan Medical Council (JMC); one as a Consultant (certified in 2012) and the other as a Specialist (certified in 2023). To assess the agreement between the two raters, Cohen’s κ statistic was utilized. The evaluation was based on the CLEAR tool [20]. Each generated response was assessed for the five attributes (Completeness, Lack of false information, Evidence support, Appropriateness, and Relevance), each on a 5-point Likert scale ranging from 5 (excellent) to 1 (poor) [20]. The assessment was preceded by a joint discussion among the three authors to determine the criteria for optimal answers in light of the CLEAR tool guidelines [20].

Range and randomization of microbiology topics tested

This study’s broad topic was diagnostic clinical microbiology laboratory. This included intra-subject variability with the creation of case scenarios based on the intentional focus to represent distinct aspects of this healthcare field. Specifically, 10 topics were selected as follows: (1) The implications of isolating a non-pathogen parasite in ova and parasite (O&P) examination, with possible indication of fecal contamination [22]; (2) The adoption of minimum inhibitory concentration (MIC) determination via broth microdilution as the standard approach for assessing colistin susceptibility [23]; (3) Resistance of methicillin-resistant *Staphylococcus aureus* (MRSA) to all beta-lactam antibiotics [24]; (4) The intrinsic resistance of Enterococci to clindamycin [25]; (5) The utilization of simple direct techniques (colonial morphology and germ tube testing) for identifying *Candida albicans* [26]; (6) The interpretation of urine culture results for the diagnosis of urinary tract infection (UTI) [27]; (7) The identification of *Brucella* spp. infection in blood specimens through biochemical and serotyping testing, with special consideration of the safety issues [28]; (8) Interpretation of threshold cycle (Ct) values in multiplex real-time polymerase chain reaction (PCR) testing for viral respiratory pathogens, along with associated QC issues [29]; (9) The significance of assessing sample quality before sputum culture [30]; and (10) The identification of *Salmonella enterica* and its serotyping based on the Kaufmann-White classification [31]. The selection of topics for testing the two ChatGPT models was non-randomized, deliberately focusing on scenarios commonly encountered in clinical microbiology laboratories, particularly in Jordan.

Statistical and data analysis

The statistical analysis in this study was conducted using IBM SPSS Statistics for Windows, Version 26 (IBM Corp. Armonk, NY). The level of statistical significance was set at P<.05.

To test the mean differences in paired observations, the paired t-test was employed, based on the normality of data distribution as confirmed using the Shapiro-Wilk test. To evaluate the variability across different items within the CLEAR tool in each ChatGPT model, the related samples Friedman’s two-way analysis of variance by ranks was used.

For the evaluation of inter-rater reliability upon comparing the content generated by both ChatGPT models, Cohen’s κ statistic was used as an approach to measure the level of agreement between the two independent raters. The interpretation of Cohen’s κ values was categorized as follows: values less than 0.20 indicated poor agreement, 0.21 to 0.40 indicated fair agreement, 0.41 to 0.60 indicated moderate agreement, 0.61 to 0.80 indicated good agreement, and 0.81 to 1.00 indicated excellent agreement [32].

The final CLEAR scores were based on the average of the two raters’ scores. For descriptive interpretation of the CLEAR scores as an indication of the quality of the generated content (sum of scores for the five items divided by 5), the scores were classified into the following categories: CLEAR scores of 1-1.79 were classified as “poor”; 1.80-2.59 as “satisfactory”; 2.60-3.39 as “good”; 3.40-4.19 as “very good”; and 4.20-5.00 as “excellent” [20].

Data availability statement

The complete phrasing of the queries and ChatGPT responses are publicly available together with the manuscript draft through the Open Science Framework (OSF) public data repository using the following link: https://osf.io/92uvz/

## Results

ChatGPT-4 outperformed ChatGPT-3.5 across the 10 queries

A consistent and statistically significant agreement between the two raters was observed upon comparisons made per each CLEAR item for both ChatGPT models. For ChatGPT-3.5 and ChatGPT-4, Cohen’s κ values indicated statistically significant fair to good inter-rater agreement (Table 1).

**Table 1:** The inter-rater agreement upon assessing ChatGPT-3.5 versus ChatGPT-4 output stratified per each CLEAR item. C: completeness; L: lack of false information; E: evidence support; A: appropriateness, R: relevance; SD: standard deviation

CLEAR item	Rater 1	Rater 2	Average score	Cohen’s κ	Asymptotic standard error, approximate T	P value
ChatGPT-3.5	Mean±SD	Mean±SD	Mean±SD			
C	2.7±0.823	2.6±1.075	2.65±0.91	0.595	0.173, 3.505	<0.001
L	2.9±1.729	2.9±1.595	2.9±1.65	0.737	0.157, 4.405	<0.001
E	2.8±1.687	2.7±1.418	2.75±1.53	0.605	0.176, 3.619	0.001
A	2.6±1.174	2.9±1.197	2.75±1.14	0.351	0.197, 2.115	0.034
R	2.1±0.876	2.2±0.632	2.15±0.71	0.492	0.212, 2.562	0.010
ChatGPT-4						
C	3.3±1.059	3.5±0.972	3.4±0.99	0.701	0.189, 3.705	<0.001
L	3.2±1.619	3.6±1.265	3.4±1.43	0.487	0.171, 3.078	0.002
E	3.3±1.567	3.5±1.179	3.4±1.35	0.506	0.168, 3.595	<0.001
A	2.9±1.197	3.5±1.269	3.2±1.18	0.359	0.189, 2.239	0.025
R	2.4±1.174	2.9±1.287	2.65±1.16	0.294	0.141, 2.545	0.011

Out of 50 pairwise comparisons between the two ChatGPT models based on the average CLEAR scores, ChatGPT-4 scored higher than ChatGPT-3.5 in 31 comparisons (62.0%), were equal in 18 comparisons (36.0%), while ChatGPT-3.5 scored higher than ChatGPT-4 in only a single encounter (2.0%, Table 2).

**Table 2:** Pairwise comparisons between the two ChatGPT models tested across the CLEAR items. C: completeness; L: lack of false information; E: evidence support; A: appropriateness, R: relevance. The average scores were calculated by the sum of the two raters’ scores divided by 2. The comparisons were based on the average of the two raters’ scores for each CLEAR item for each query (Q).

Query	Model	Average C	Average L	Average E	Average A	Average R
Q1	ChatGPT-3.5	2.5	1.5	1.5	1	2
	ChatGPT-4	3	1.5	1.5	1.5	1.5
Q2	ChatGPT-3.5	2	1	1	2	1
	ChatGPT-4	2	1.5	1.5	2	1
Q3	ChatGPT-3.5	3	3	3	3	2
	ChatGPT-4	3	3.5	3.5	3.5	2.5
Q4	ChatGPT-3.5	2	1	1	1.5	1.5
	ChatGPT-4	3	2	2	2	2.5
Q5	ChatGPT-3.5	4	5	4.5	4	3
	ChatGPT-4	5	5	4.5	5	4.5
Q6	ChatGPT-3.5	3	4	3	2.5	2
	ChatGPT-4	3	4	3	2.5	2
Q7	ChatGPT-3.5	4	5	5	4.5	3.5
	ChatGPT-4	5	5	5	4.5	4
Q8	ChatGPT-3.5	1.5	1	1	2	2
	ChatGPT-4	4	2.5	4	3	4
Q9	ChatGPT-3.5	1.5	3.5	3.5	3.5	2
	ChatGPT-4	2.5	4	4	4	2
Q10	ChatGPT-3.5	3	4	4	3.5	2.5
	ChatGPT-4	3.5	5	5	4	2.5

Performance of ChatGPT-3.5 and ChatGPT-4 per each CLEAR item

**Figure 1:**
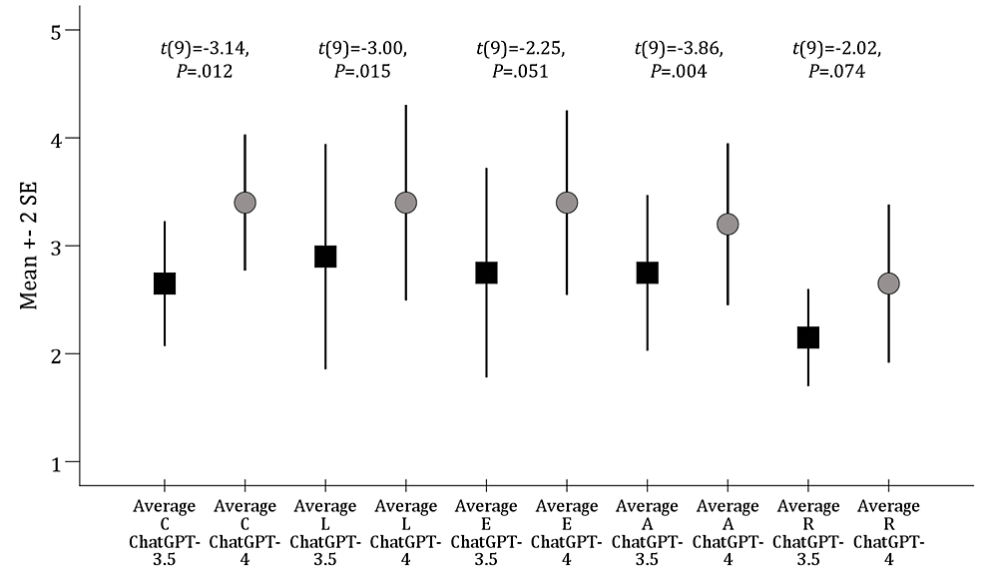
Comparisons of the average performance of ChatGPT-3.5 versus ChatGPT-4 per each CLEAR item. SE: standard error of the mean, C: completeness; L: lack of false information; E: evidence support; A: appropriateness, R: relevance. The squares indicate ChatGPT-3.5 means, while the circles indicate ChatGPT-4 means. P values were based on the paired t-test. The average scores were calculated by the sum of the two raters’ scores divided by 2.

The overall CLEAR scores based on the sum of mean values across the 10 queries were used to compare the performance of each ChatGPT model across the five CLEAR items. ChatGPT-4 outperformed ChatGPT-3.5 in the “Appropriateness”, “Lack of false information”, and “Completeness” items with the difference showing a statistical significance (Figure 1).

Within-model variability in performance per CLEAR items

Upon comparing the performance of each ChatGPT model, differences were observed based on the CLEAR items. In ChatGPT-3.5, despite the variability in performance, with the highest score in “Lack of false knowledge” item and the lowest score in the “Relevance” item, this difference lacked statistical significance (χ24=4.907, P=.297). On the other hand, ChatGPT-4 showed the best performance in completeness, lack of false knowledge, and evidence-based content, while the lowest performance was in relevance (χ24=9.863, P=0.043).

Comparison of ChatGPT-3.5 versus ChatGPT-4 performance per query

Despite falling in the same descriptive CLEAR category based on the overall performance in which both models showed “good” performance, ChatGPT-4 performance was better than ChatGPT-3.5 with the difference showing a statistical significance (mean: 2.64±1.06 vs. 3.21±1.05, P=.012).

Per topic, the lowest performance was observed for queries that involved AST assessment with both models showing only a satisfactory level of performance, while the highest performance was in the microbial identification category with very good performance (Table 3).

**Table 3:** Assessment of ChatGPT models’ performance per topic and the overall performance across topics. O&P: Ova and parasite examination; AST: antimicrobial susceptibility testing; MRSA: methicillin-resistant *Staphylococcus aureus*; UTI: urinary tract infection; PCR: polymerase chain reaction; ID: microbial identification; Dx: diagnostic approach; CLEAR: Completeness, Lack of false information, Evidence support, Appropriateness, and Relevance. The average scores were calculated by the sum of the two raters’ scores divided by 2.

CASE	Query classification	Average CLEAR score for ChatGPT-3.5	Average CLEAR score for ChatGPT-4	t-test
Average performance in ID		3.4 (Very good)	3.83 (Very good)	t(3)=-3.087, P=0.054
Q1 (O&P examination)	ID	1.7 (Poor)	1.8 (Satisfactory)	
Q5 (*Candida albicans* identification)	ID	4.1 (Very good)	4.8 (Excellent)	
Q7 (*Brucella* spp. identification)	ID	4.4 (Excellent)	4.7 (Excellent)	
Q10 (*Salmonella enterica* identification)	ID	3.4 (Very good)	4.0 (Very good)	
Average performance in AST		1.87 (Satisfactory)	2.37 (Satisfactory)	t(2)=-1.387, P=0.300
Q2 (AST for colistin)	AST	1.4 (Poor)	1.6 (Poor)	
Q3 (MRSA resistance to all beta-lactams)	AST	2.8 (Good)	3.2 (Good)	
Q4 (Enterococci resistance to clindamycin)	AST	1.4 (Poor)	2.3 (Satisfactory)	
Average performance in Dx		2.4 (Satisfactory)	3.2 (Good)	t(2)=-2.402, P=0.138
Q6 (Laboratory diagnosis of UTI)	Dx	2.9 (Good)	2.9 (Good)	
Q8 (Interpretation of real-time PCR testing for respiratory viruses/atypical bacteria)	Dx	1.5 (Poor)	3.5 (Very good)	
Q9 (Sputum quality assessment for microbiologic culture)	Dx	2.8 (Good)	3.3 (Good)	
Overall performance across the three categories		2.64 (Good)	3.21 (Good)	t(9)=-3.143, P=0.012

## Discussion

The practice of diagnostic clinical microbiology requires meticulous diligence. Therefore, it is important to continuously evaluate the performance of AI models in such a highly precise healthcare discipline. The reliability of AI-generated health information may prove useful or even essential to health professionals including microbiologists in the near future [33-36]. While AI-based models such as ChatGPT showed promising perspectives in various healthcare disciplines, their current limitations necessitate continued development and rigorous evaluation to ensure their reliability and accuracy in different clinical settings [2,6].

The current study employed a novel tool referred to as “CLEAR”, which is helpful for standardizing the evaluation of information generated by AI-based models such as ChatGPT [20]. By identifying knowledge gaps, information inaccuracies, ambiguities, and biases generated by these models, the CLEAR tool provides a framework to systematically assess health query responses. Subsequently, the findings can shed light on areas that need to be improved in these AI-based tools.

In the current study, the findings pointed to the variable performance of both ChatGPT models. Specifically, while satisfactory at minimum in response to the majority of queries, certain ChatGPT responses contained critical errors, highlighting the risk of possible detrimental outcomes if such content is used in clinical decision-making.

The current study evaluated the performance of both ChatGPT-3.5 and ChatGPT-4, with the latter demonstrating superior capabilities based on the finding of higher CLEAR scores for the advanced model. Despite the subjectivity in the assessment of ChatGPT performance in this study, the fair to good consistency of inter-rater agreement across all the CLEAR items for both models supports the credibility of the methodologic approach used.

In this study, ChatGPT-4 in particular, exhibited higher performance ratings across all the CLEAR items, suggesting a significant improvement in the evolution of this large language model. This pattern was also observed across a wide range of healthcare-related studies. For example, Hirosawa et al. showed that ChatGPT-4 achieved the correct diagnosis in the differential diagnosis lists compared to human physicians with better performance compared to ChatGPT-3.5 [37]. Additionally, Teebagy et al. demonstrated the superior performance of ChatGPT-4 compared to ChatGPT-3.5 in the Ophthalmology Knowledge Assessment Program examination [38]. Moreover, Massey et al. highlighted the superior performance of ChatGPT-4 compared to ChatGPT-3.5 regarding the ability to answer orthopedic resident assessment examination questions [39]. Furthermore, a recent study by Moshirfar et al. showed that ChatGPT-4 had a significant performance advantage compared to ChatGPT-3.5 and human professionals in answering ophthalmology StatPearls queries [40].

Despite the inferior performance of the freely accessible version (ChatGPT-3.5), this model showed strength in terms of providing accurate information, particularly in the “Lack of false knowledge” item of the CLEAR scale. However, the low performance in the “Relevance” item highlights the need for enhancements in contextual understanding in this model. Similarly, ChatGPT-4 had its worst performance in the “Relevance” item as well, indicating the general need to enhance ChatGPT’s ability to generate pertinent, contextually appropriate responses. This tendency to include unnecessary content might undermine the utility of ChatGPT responses in healthcare practice. On the other hand, the performance of ChatGPT-4 in the “Completeness”, “Lack of false knowledge”, and “Evidence-based output” was noteworthy, suggesting the ability of this advanced model to provide comprehensive, accurate, and evidence-based health information.

On the contrary, a closer look at the fine granularity of the ChatGPT-generated content revealed certain deficiencies. Critical aspects like the necessity of QC measures and the urgency of reporting critical results were overlooked at certain encounters. Such deficiencies are considered critical since they could significantly impact patient outcomes [41,42]. Additionally, both ChatGPT models showed limitations in answering queries related to AST. Notably, both models incorrectly suggested clindamycin as an option for treating enterococcal infections and failed to mention the appropriate standard method for evaluating colistin susceptibility among *Acinetobacter* isolates. Such inaccuracies could lead to ineffective treatment choices with negative patient outcomes. Notably, the suboptimal performance of ChatGPT-3.5 in the interpretation of the real-time multiplex PCR case suggests a limitation in handling complex diagnostic processes, which is a key element in modern clinical microbiology laboratory practice. In this study, the solution to this deficiency can be based on the ongoing refinement of AI-based models, as evidenced by the markedly enhanced capabilities of ChatGPT-4 in the same case scenario. The below-average performance of ChatGPT-3.5 in medical microbiology was highlighted in a recent study that compared the performance of this AI model to human students [11].

Previous studies have clearly outlined the possible biases and factual inaccuracies in ChatGPT-generated content in different healthcare-related contexts [2,6,43-47]. A possible explanation for this obvious trend in healthcare might be related to the limited access to copyrighted material and annually updated guidelines with the limitation of knowledge limit based on the training data of these AI-based models [2,14]. Thus, a challenge that needs to be addressed is the need for dynamic incorporation of information into AI algorithms to ensure the generation of up-to-date and accurate content.

Finally, the current study was limited by several shortcomings that warrant careful consideration. This included the subjective, descriptive nature of the evaluation process compounded by varying expertise levels of the raters. This was reflected in relatively low κ values for the relevance and appropriateness items. Additionally, constructing the case scenarios, though representative of common clinical microbiology laboratory scenarios, may lack the randomization needed to mitigate selection bias. Moreover, the small sample size, comprising only 10 queries, is acknowledged, yet it can form the basis for future, more comprehensive studies addressing AI-based models’ utility in clinical microbiology, including both common and rare case scenarios. Additionally, the exclusive use of the English language in the study may not reflect ChatGPT performance variations in other linguistic or cultural contexts.

## Conclusions

To the best of our knowledge, the current study was the first attempt to assess ChatGPT performance in diagnostic microbiology using a standardized approach. While both ChatGPT models have shown satisfactory results in some cases, their application in clinical microbiology currently remains in infancy, given the need for precision in this field of healthcare practice. A primary concern was the common encounter of irrelevant content in responses in both models. The findings indicated that AI-based models like ChatGPT are advancing, with ChatGPT-4 demonstrating superior performance over ChatGPT-3.5 in clinical microbiology. However, improvements are still needed through continuous refinement and targeted training with particular importance on improving relevance and contextual accuracy. Additionally, the development of AI-based models specifically designed and trained for healthcare purposes can be another approach to reap the benefits of AI with high precision.

Finally, it is difficult to assign ChatGPT performance in this study to a specific label (e.g., above average, good, or mediocre) considering the limitations of the study. Nevertheless, the findings of this study highlighted both the promising potential and concerning challenges of integrating AI-based models into clinical microbiology practice. On the positive side, AI-based models could enhance the workflow in clinical laboratories, facilitating the design of reports’ layouts and detailing laboratory procedures. However, the study results also raised valid concerns regarding both ChatGPT models, particularly highlighting a lack of adequate emphasis on QC measures, which is a critical aspect in clinical settings.

## References

[1] Bajwa J, Munir U, Nori A, Williams B (2021). Artificial intelligence in healthcare: Transforming the practice of medicine. Future Healthc J.

[2] Sallam M (2023). ChatGPT utility in healthcare education, research, and practice: Systematic review on the promising perspectives and valid concerns. Healthcare (Basel).

[3] Jens K, Yonghui W, Gregor S, Jan E, Jiang B (2023). An opinion on ChatGPT in health care—Written by Humans Only. J Nucl Med.

[4] (2023). ChatGPT. (2023). https://openai.com/chatgpt.

[5] Sallam M (2023). Bibliometric top ten healthcare related ChatGPT publications in Scopus, Web of Science, and Google Scholar in the first ChatGPT anniversary [PREPRINT]. JMIR Preprints.

[6] Li J, Dada A, Kleesiek J, Egger J (2023). ChatGPT in healthcare: A taxonomy and systematic review [PREPRINT]. medRxiv.

[7] Irfan B, Yaqoob A (2023). ChatGPT's Epoch in rheumatological diagnostics: A critical assessment in the context of Sjögren's Syndrome. Cureus.

[8] Köroğlu EY, Fakı S, Beştepe N (2023). A novel approach: Evaluating ChatGPT's utility for the management of thyroid nodules. Cureus.

[9] Oca MC, Meller L, Wilson K (2023). Bias and inaccuracy in AI chatbot ophthalmologist recommendations. Cureus.

[10] Puladi B, Gsaxner C, Kleesiek J, Hölzle F, Röhrig R, Egger J (2023). The impact and opportunities of large language models like ChatGPT in oral and maxillofacial surgery: a narrative review [IN PRESS]. Int J Oral Maxillofac Surg.

[11] Sallam M, Al-Salahat K (2023). Below average ChatGPT performance in medical microbiology exam compared to university students. Front Educ.

[12] Hasani N, Farhadi F, Morris MA (2022). Artificial intelligence in medical imaging and its impact on the rare disease community: Threats, challenges and opportunities. PET Clin.

[13] Sarker IH (2022). AI-based modeling: Techniques, applications and research issues towards automation, intelligent and smart systems. SN Comput Sci.

[14] Khan B, Fatima H, Qureshi A, Kumar S, Hanan A, Hussain J, Abdullah S (2023). Drawbacks of artificial intelligence and their potential solutions in the healthcare sector. Biomed Mater Devices.

[15] Davenport T, Kalakota R (2019). The potential for artificial intelligence in healthcare. Future Healthc J.

[16] Najjar R (2023). Redefining radiology: A review of artificial intelligence integration in medical imaging. Diagnostics.

[17] Försch S, Klauschen F, Hufnagl P, Roth W (2021). Artificial intelligence in pathology. (Article in German). Dtsch Arztebl Int.

[18] Shelke YP, Badge AK, Bankar NJ (2023). Applications of artificial intelligence in microbial diagnosis. Cureus.

[19] Sallam M, Barakat M, Sallam M (2023). METRICS: Establishing a preliminary checklist to standardize design and reporting of artificial intelligence-based studies in healthcare [PREPRINT]. JMIR Preprints.

[20] Sallam M, Barakat M, Sallam M (2023). Pilot testing of a tool to standardize the assessment of the quality of health information generated by artificial intelligence-based models. Cureus.

[21] Meskó B (2023). Prompt engineering as an important emerging skill for medical professionals: Tutorial. J Med Internet Res.

[22] Alzate JF, Toro-Londoño M, Cabarcas F, Garcia-Montoya G, Galvan-Diaz A (2020). Contrasting microbiota profiles observed in children carrying either Blastocystis spp. or the commensal amoebas Entamoeba coli or Endolimax nana. Sci Rep.

[23] Chew KL, La MV, Lin RT, Teo JW (2017). Colistin and polymyxin B: Susceptibility testing for carbapenem-resistant and mcr-positive Enterobacteriaceae: Comparison of Sensititre, MicroScan, Vitek 2, and Etest with broth microdilution. J Clin Microbiol.

[24] Pinho MG, de Lencastre H, Tomasz A (2001). An acquired and a native penicillin-binding protein cooperate in building the cell wall of drug-resistant staphylococci. Proc Natl Acad Sci U S A.

[25] Singh KV, Weinstock GM, Murray BE (2002). An Enterococcus faecalis ABC homologue (Lsa) is required for the resistance of this species to clindamycin and quinupristin-dalfopristin. Antimicrob Agents Chemother.

[26] Baron EJ (2001). Rapid identification of bacteria and yeast: Summary of a National Committee for Clinical Laboratory Standards proposed guideline. Clin Infect Dis.

[27] Schmiemann G, Kniehl E, Gebhardt K, Matejczyk MM, Hummers-Pradier E (2010). The diagnosis of urinary tract infection: A systematic review. (Article in German). Dtsch Arztebl Int.

[28] Di Bonaventura G, Angeletti S, Ianni A, Petitti T, Gherardi G (2021). Microbiological laboratory diagnosis of human brucellosis: An overview. Pathogens.

[29] Bouzid D, Vila J, Hansen G, Manissero D, Pareja J, Rao SN, Visseaux B (2021). Systematic review on the association between respiratory virus real-time PCR cycle threshold values and clinical presentation or outcomes. J Antimicrob Chemother.

[30] García-Vázquez E, Marcos MA, Mensa J (2004). Assessment of the usefulness of sputum culture for diagnosis of community-acquired pneumonia using the PORT predictive scoring system. Arch Intern Med.

[31] Wattiau P, Boland C, Bertrand S (2011). Methodologies for Salmonella enterica subsp. enterica subtyping: Gold standards and alternatives. Appl Environ Microbiol.

[32] Sallam M, Salim NA, Al-Tammemi AB (2023). ChatGPT output regarding compulsory vaccination and COVID-19 vaccine conspiracy: A descriptive study at the outset of a paradigm shift in online search for information. Cureus.

[33] Kaneda Y, Takita M, Hamaki T, Ozaki A, Tanimoto T (2023). ChatGPT's potential in enhancing physician efficiency: A Japanese case study. Cureus.

[34] Sultan I, Al-Abdallat H, Alnajjar Z, Ismail L, Abukhashabeh R, Bitar L, Abu Shanap M (2023). Using ChatGPT to predict cancer predisposition genes: A promising tool for pediatric oncologists. Cureus.

[35] Alan R, Alan BM (2023). Utilizing ChatGPT-4 for providing information on periodontal disease to patients: A DISCERN quality analysis. Cureus.

[36] Chinnadurai S, Mahadevan S, Navaneethakrishnan B, Mamadapur M (2023). Decoding applications of artificial intelligence in rheumatology. Cureus.

[37] Hirosawa T, Kawamura R, Harada Y (2023). ChatGPT-generated differential diagnosis lists for complex case-derived clinical vignettes: Diagnostic accuracy evaluation. JMIR Med Inform.

[38] Teebagy S, Colwell L, Wood E, Yaghy A, Faustina M (2023). Improved performance of ChatGPT-4 on the OKAP Examination: A comparative study with ChatGPT-3.5. J Acad Ophthalmol.

[39] Massey PA, Montgomery C, Zhang AS (2023). Comparison of ChatGPT-3.5, ChatGPT-4, and orthopaedic resident performance on orthopaedic assessment examinations. J Am Acad Orthop Surg.

[40] Moshirfar M, Altaf AW, Stoakes IM, Tuttle JJ, Hoopes PC (2023). Artificial intelligence in ophthalmology: A comparative analysis of GPT-3.5, GPT-4, and human expertise in answering StatPearls questions. Cureus.

[41] Carey RB, Bhattacharyya S, Kehl SC, Matukas LM, Pentella MA, Salfinger M, Schuetz AN (2018). Practical guidance for clinical microbiology laboratories: Implementing a quality management system in the medical microbiology laboratory. Clin Microbiol Rev.

[42] Genzen JR, Tormey CA (2011). Pathology consultation on reporting of critical values. Am J Clin Pathol.

[43] Abu Hammour K, Alhamad H, Al-Ashwal FY, Halboup A, Abu Farha R, Abu Hammour A (2023). ChatGPT in pharmacy practice: a cross-sectional exploration of Jordanian pharmacists' perception, practice, and concerns. J Pharm Policy Pract.

[44] Bhattacharyya M, Miller VM, Bhattacharyya D, Miller LE (2023). High rates of fabricated and inaccurate references in ChatGPT-generated medical content. Cureus.

[45] Gravel J, D’Amours-Gravel M, Osmanlliu E (2023). Learning to fake it: Limited responses and fabricated references provided by ChatGPT for medical questions. Mayo Clinic Proceedings: Digital Health.

[46] Cinar C (2023). Analyzing the performance of ChatGPT about osteoporosis. Cureus.

[47] Jeyaraman M, Balaji S, Jeyaraman N, Yadav S (2023). Unraveling the ethical enigma: Artificial intelligence in healthcare. Cureus.

